# Ultrasonically-assisted synthesis of CeO_2_ within WS_2_ interlayers forming type II heterojunction for a VOC photocatalytic oxidation

**DOI:** 10.1016/j.ultsonch.2022.106245

**Published:** 2022-11-28

**Authors:** Esmail Doustkhah, Ramin Hassandoost, Negar Yousef Tizhoosh, Mohamed Esmat, Olga Guselnikova, M. Hussein N. Assadi, Alireza Khataee

**Affiliations:** a Koç University Tüpraş Energy Center (KUTEM), Department of Chemistry, Koç University, 34450 Istanbul, Turkey; b Research Laboratory of Advanced Water and Wastewater Treatment Processes, Department of Applied Chemistry, Faculty of Chemistry, University of Tabriz, 51666-16471 Tabriz, Iran; c International Center for Materials Nanoarchitechtonics (MANA), National Institute for Materials Science (NIMS), 1-1 Namiki, Tsukuba, Ibaraki 305-0044, Japan; d Materials Science and Nanotechnology Department, Faculty of Postgraduate Studies for Advanced Sciences (PSAS), Beni-Suef University (BSU), Beni-Suef 62511, Egypt; e Research School of Chemistry & Applied Biomedical Sciences, National Research Tomsk Polytechnic University, Lenin Avenue 30, Tomsk 634050, Russia; f RIKEN Center for Emergent Matter Science, 2-1 Hirosawa, Wako, Saitama 351-0198, Japan; g Department of Environmental Engineering, Gebze Technical University, 41400 Gebze, Turkey

**Keywords:** CeO_2_, Formic acid oxidation, Heterostructure, Layered WS_2_, Ultrasonically intercalated CeO_2_, Photocatalytic oxidation

## Abstract

Here, we investigate the band structure, density of states, photocatalytic activity, and heterojunction mechanism of WS_2_ with CeO_2_ (CeO_2_@WS_2_) as a photoactive heterostructure. In this heterostructure, CeO_2_′s growth within WS_2_ layers is achieved through ultrasonicating WS_2_ and intercalating CeO_2_′s precursor within the WS_2_ interlayers, followed by hydrothermal treatment. Through a set of density functional calculations, we demonstrate that CeO_2_ and WS_2_ form an interface through a covalent bonding that can be highly stable. The electrochemical impedance spectroscopy (EIS) found that the CeO_2_@WS_2_ heterostructure exhibits a remarkably higher conductivity (22.23 mS cm^−2^) compared to either WS_2_ and CeO_2_, assignable to the interface in CeO_2_@WS_2_. Furthermore, in a physically mixed CeO_2_-WS_2_ where the interaction between particles is noncovalent, the resistance was significantly higher (0.67 mS cm^−2^), confirming that the heterostructure in the interface is covalently bonded. In addition, Mott-Schottky and the bandgap measurements through Tauc plots demonstrate that the heterojunction in CeO_2_ and WS_2_ is type II. Eventually, the CeO_2_@WS_2_ heterostructure indicated 446.7 µmol g ^−1^ CO_2_ generation from photocatalytic oxidation of a volatile organic compound (VOC), formic acid, compared to WS_2_ and CeO_2_ alone.

## Highlights

• Utilisation of ultrasonically exfoliated 2D WS_2_ as hard template for CeO_2_ growth.
• Simple hydrothermal approach for synthesising CeO_2_@WS_2_ heterojunction.
• Formation of efficient type (II) heterojunction between WS_2_ and CeO_2_.
• Proving the Ce-S bonding by XRD and density functional calculations.
• Formic acid photocatalytic oxidation activity, producing 446.7 µmol g ^−1^ CO_2_.

## Graphical abstract

**Figure:**
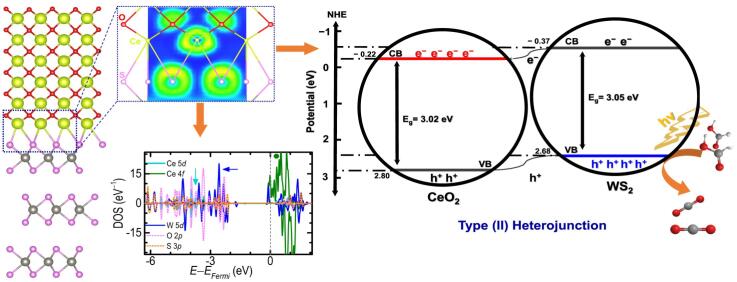

## 1 Introduction

Solar-driven light in catalysis, *i.e.* photocatalysis, has a universal appeal for use as a free energy source, which can be incorporated into many applications such as fine chemicals production, energy carriers generation, and pollutants removal [1], [2], [3], [4]. Although many semiconductors have suitable energy band gaps that fall in the UV–vis range for photocatalysis, their photoactivity is often less efficient due to the improper band edge (valence band or conduction band) alignment against the potential redox energy level of the target reaction [5], [6]. Although there are several pathways for improving the photocatalytic performance in such semiconductors [7], [8], among them, a promising solution is a heterojunction within a suitable heterostructure to align the bandgap. Furthermore, the lifetimes of photogenerated electron-hole in most catalysts are short and suffer from fast recombination, which heterojunctions can further improve [9]. However, a heterostructure is only efficient if each of its semiconductor’s band edges is suitably aligned so it can eventually transfer the photogenerated electrons/holes pairs to the reacting species. Another point in a successful heterostructure is the crystalline structure at the interface of the junction [10]. The electron transfer in the boundary will be less efficient if there is no suitable junction due to mismatching, cracked or loose interface. So far, the 2D dichalcogenide WS_2_ forms promising heterostructures with other photoactive oxides and semiconductors such as TiO_2_ [11], MoS_2_ [12], SnS [13], and CuO [14]. However, the heterostructure of CeO_2_ with WS_2_ in photocatalysis is rarely investigated [15]. Likewise, cerium oxide has attractive crystal and photocatalytic properties and has been found suitable for the heterostructure through materials nanoarchitecture [16], [17], [18].

Intercalation of (organo)metallic precursors in 2D layered materials’ interlayers is one of the pivotal approaches to synthesising heterostructures with oriented and uniform growth [19], [20]. For such cases, the exfoliation of bulk structures into few/multilayer structures is crucial to intercalate the heterostructure’s precursor. Among the several methods, ultrasound waves as a powerful tool for synthesis of nanomaterials [21], can be also utilised for exfoliating bulk layers are a popular and efficient pathway [22], [23], [24], [25]. Likewise, WS_2_ is a 2D material that may act as a hard template while it can generate a heterojunction with the grown heterostructure [26]. However, in an efficient heterojunction, along with its well-synthesised architecture, the band edges and the energy bandgaps of the composing compounds must match one another to generate an enhanced photocatalytic activity [27], [28]. Therefore, investigating such parameters in parallel with the synthesis method is vital to reach an optimal photocatalysis result.

Here, we have found that the heterojunction of CeO_2_ with the WS_2_ nanosheets leads to a type II heterojunction which generates a remarkable enhancement in the photocatalytic activity of formic acid decomposition with respect to pure WS_2_ and CeO_2_. We also demonstrated that the heterostructure in the interface creates a chemical bonding that makes the heterostructure more efficient, stable, and uniform. The Raman spectroscopy and X-ray diffraction results showed the formation of Ce-S bonding in the CeO_2_@WS_2_. This heterostructure exhibits better photocatalytic activity, 1.8 times of WS_2_ and 2.2 times of CeO_2_. It also reveals sustainability in the UV range toward photooxidation of volatile organic compound (VOC)—formic acid.

## 2 Materials and methods

### 2.1 Synthesis of samples

The detailed synthesis procedure of the CeO_2_@WS_2_ catalyst is reported in our previous work [15]. In brief, the WS_2_ nanoflakes were obtained by ultrasound-assisted exfoliation (Sonica ultrasound bath, 300 W, Italy) of bulk WS_2_ (Sigma-Aldrich, 99 %) in DMF (Merck, >99 %) for 2 h. After centrifuging the sample, the supernatant was removed, washed with EtOH, and dried at room temperature overnight. For synthesising CeO_2_@WS_2_, 0.1 g of the synthesised WS_2_ nanoflakes were added to 60 mL 0.1 mol/L Ce(NO_3_).6H_2_O (Merck, >99.99 %) in aqueous solution and sonicated for 3 h. After that, the mixture was added to a Na_3_PO_4_·12H_2_O (Merck, >99.99 %) solution (0.005 mol/L, 20 mL) and was stirred for 30 min. The final mixture was poured into a 100 mL hydrothermal autoclave and kept at 220 °C for 12 h. After cooling the autoclave, the liquid was removed by centrifuge and the remaining was washed with EtOH and deionised water and dried at 60 °C. The synthesis route for pure CeO_2_, used in this work, was similar to the mentioned approach without adding WS_2_ nanoflakes.

### 2.2 Characterisation

Scanning electron microscopy (SEM) images were obtained using a TESCAN MIRA_3_ microscope (TESCAN Ltd, Czech Republic). X-ray diffraction (XRD) patterns were recorded with a SmartLab X-ray diffractometer (Rigaku Co., Japan, *via* a Cu Kα radiation source). Raman spectroscopy was recorded by the ANDOR Kymera-328i apparatus (Andor Tech. Ltd., United Kingdom, at 532 nm excitation wavelength with 50 × objective, in the range of 100–2000 cm^−1^). Transmission electron microscopy (TEM) and TEM-based elemental mapping and electron dispersive spectroscopy (TEM-EDS) were all recorded by a JEOL 2100 instrument coupled to an energy-dispersive X-ray analyser (EDX, JEOL Ltd, Japan).

### 2.3 (Photo)electrochemical measurements

Fluorine-doped tin oxide (FTO) glass with an area of 1.0 × 1.0 cm^2^ was used as a substrate for the preparation of the working electrode. First, 20 mg of each catalyst sample was dispersed for 45 min in a solution including 1 mL of DI and 3 mL of isopropanol. Then, the FTO was washed and activated by diluted acid (HNO_3_). 80 µL of stable dispersed catalyst was slowly drop-casted on the FTO and heated to 120 °C on a hotplate for 15 min.

An OrigaFlex-OGF01A Potentiostat/Galvanostat (France) was used to record the electrochemical analysis data. The electrochemical setup included a 0.5 mol/L Na_2_SO_4_ electrolyte, an FTO-based working electrode, a platinum plate with an area of 2.0 × 1.0 cm^2^ as the counter electrode, and a reference electrode of saturated calomel electrode (SCE). Prior to each electrochemical test, the electrolyte solution was degassed by nitrogen bubbling for 20 min.

The electrochemical properties of samples were tested through the photocurrent test by cyclic chronoamperometry (CA) under both dark and light conditions, the EIS, and Mott-Schottky plots to determine the energy of flat band (E_fb_). Mott-Schottky plots were recorded at a frequency of 1000 Hz and the voltage range of −1 ∼ +1 V *vs* SCE in dark. The EIS results were obtained using an AC voltage of ± 5 mV at open circuit potential (OCP) and 300 mV *vs* SCE in a frequency range from 100 kHz to 100 mHz. Photocurrent behaviours were tested under consecutive light on–off cycles (each cycle time: 100 s) for 600 s at 1000 mV *vs* SCE.

### 2.4 Computational settings

Spin polarised density functional calculations were performed using VASP code [29], [30] with the projector augmented wave method technique [31]. The electronic correlation-exchange energy was approximated with general gradient approximation within the Perdew-Burke-Ernzerhof formalism [32], [33]. The energy cutoff value and the fast Fourier transform mesh were generated by setting the precision key to *Accurate*. *Ad hoc* Hubbard terms, based on the Dudarev implementation [34], were added to Ce and W 5*d* electrons to account for the strong correlation. For Ce, *U* and *J* values were 4.20 eV and 0.00 eV, respectively. For W, *U* and *J* values were 2.87 eV and 0.00 eV, respectively. These values were reported to improve the electronic description of CeO_2_ [35] and WS_2_ [36], respectively. Van der Waals dispersion energy correction was applied based on the DFT-D3 method [37]. Only Γ point was used for the Brillouin zone sampling. For geometry optimisation, in addition to the atomic coordinates, the lateral lattice parameters, *u* and *v*, were also allowed to relax to forces smaller than 0.01 eV/Å. The Bader charge analysis code [38] was used to analyse charge localisations.

### 2.5 Formic acid photocatalytic oxidation

For formic acid photooxidation, 15 mg of the catalyst was initially dispersed and sonicated for 1 min in 5 mL of a formic acid aqueous solution (5 vol%, Merck, >98 %) in a Pyrex glass test tube (34 mL) and then bubbled with O_2_ gas for 30 min. The glass tube was sealed with a rubber septum and photo-irradiated by a solar simulator (San-Ei Electric, λ > 300 nm, 1000 W m^−2^), stirring at 800 rpm. The amount of evolved CO_2_ in the headspace of the sealed tube was determined by a Shimadzu GC-2010 plus gas chromatograph equipped with a barrier ionisation discharge (BID) detector.

## 3 Results and discussion

### 3.1 Materials characterisation

**Fig. 1:**
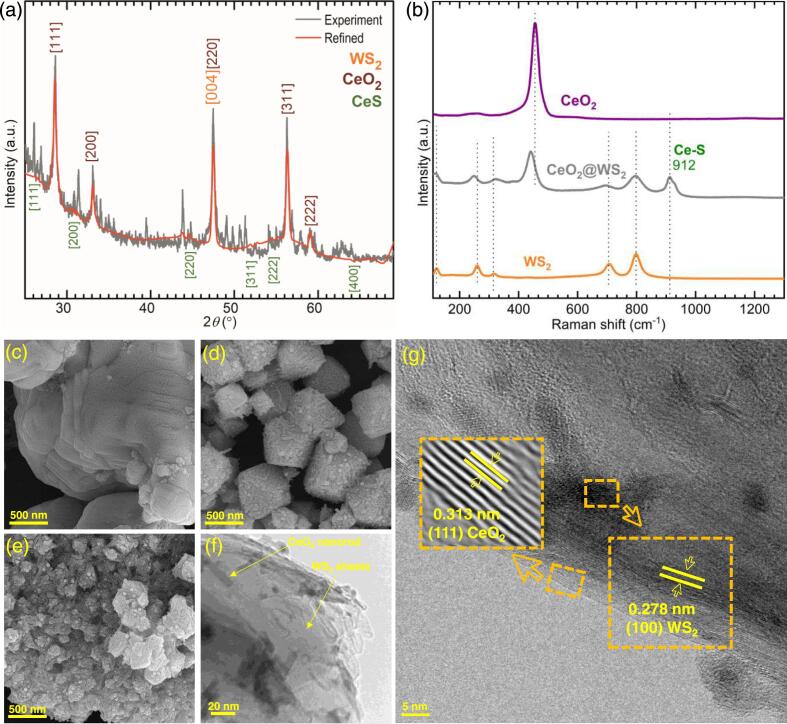
(a) Observed and refined XRD patterns for the synthesised CeO_2_@WS_2_. (b) Raman spectra of CeO_2_, WS_2_ and CeO_2_@WS_2_ heterostructure. SEM images of (c) WS_2_, (d) CeO_2_, and (e) CeO_2_@WS_2_ heterostructure. (f) Low and high magnification TEM images of the CeO_2_@WS_2_. Insets are HRTEM images of the same material.

We present the synthesis of ceria by the hydrothermal growth through intercalating Ce^4+^ in the interlayer space of WS_2_, exfoliated by sonication in an aqueous solution. According to the XRD pattern, shown in Fig. 1a, the synthesised material indicates a clear set of peaks corresponding to the CeO_2_, WS_2_ and CeS structures. Furthermore, given the non-equilibrium growth regime, intense nanostructuring and random growth of CeO_2_ crystallites, the XRD pattern appears with strong background noise and some unidentified peaks, most likely belonging to the W-S-Ce-O alloys with nonstoichiometric compositions for which we do not have a reference structure for refinement. However, we attempted to refine the identified peaks using the Rietveld refinement Fullprof Suite [39] and the Match! package [40]. With acceptable quality parameters of Bragg factor of 20.3 and a *Χ*^2^ of 7.5, the content was found to be 76.86 % (±0.03 %) CeO_2_, 7.00 % (±0.02 %) WS_2_, and 16.14 % (±0.02 %) CeS. The lattice parameters of these phases were found to be *a* = 5.414598 Å (±0.000527 Å) for the cubic CeO_2_, *a* = 2.970194 Å (±0.005109 Å) and *c* = 7.579407 Å (±0.009297 Å) for the hexagonal WS_2_, and *a* = 5.799615 Å (±0.004254 Å) for the cubic CeS. Also, the XRD pattern of physically mixed CeO_2_ and WS_2_ is shown in Fig. S1. We further investigated the changes in the vibrational modes originating from the heterojunction of CeO_2_ and WS_2_ using Raman spectroscopy (Fig. 1b). Accordingly, the peaks at 799, 801, 705, 317, 260, and 122 cm^−1^ in WS_2_′s spectra with a slight shift in some to lower wavenumbers and a decrease in the intensity have appeared again in the CeO_2_@WS_2_ heterostructure. The 456 cm^−1^ peak in CeO_2_′s spectra is characteristic of the CeO_2_ structure, which was also shifted lower to 442 cm^−1^ and decreased in intensity in the final CeO_2_@WS_2_ heterostructure. Eventually, a new peak appeared at 912 cm^−1,^ which is not originating from either WS_2_ or CeO_2_ and is assignable to the newly formed Ce-S bond [41]. Therefore, the Raman spectra prove that there can be a covalent bonding between Ce and S species in the interface of CeO_2_ and WS_2_.

As shown in Fig. 1c-g, we studied morphology and the structure of the CeO_2_-intercalated WS_2_ (CeO_2_@WS_2_) through SEM and TEM. In TEM and SEM micrographs (Fig. 1d-h), we can see an agglomeration of uniform nanorods in each particle, hinting to the successful heterostructure formation of CeO_2_ and WS_2_ within each nanorod, as all these nanorods are approximately of the same size and shape. Phase separation would have probably caused different nano-shapes for WS_2_ and CeO_2_ as these phases have different crystal symmetry. The HRTEM in the inset of Fig. 1g shows two distinct lattice structures belonging to CeO_2_ and WS_2_ within the matrix, confirming the presence of the CeO_2_@WS_2_ heterostructure. The observed and specified *d* values in the HRTEM image can be assigned to the planes with Miller indices of CeO_2_ (1 1 1) and WS_2_ (1 0 0) [42], [43], [44], indicating the interface in the heterostructures is normal to these planes. TEM-EDS of a selected area under a TEM microscope (Fig. S2) further confirms the co-presence of the related elements in a single particle, showing that the heterostructures have successfully interconnected. TEM-based elemental mapping further proves the presence of the Ce, O, W, and S elements in one particle (Fig. S2).

### 3.2 (Photo)electrochemical results

Appropriate separation of photogenerated charge carriers at the surface of particles is one of the most critical factors in photocatalytic performance. Photoluminescence (PL) is a valuable method to investigate the charge separation efficiency in materials. As reported before [45], CeO_2_ possesses a board emission band from 400 to 700 nm with the most intensity in the 470 ∼ 570 nm region. The charge transfer in the CeO_2_ originated from the electron transfer between the Ce 4*f* energy level and O 2*p* orbitals [46]. Therefore, this board peak appears due to the existence of many defect energy levels between the mentioned orbitals [47]. According to our observation in the previous report [15], a remarkably diminished CeO_2_ emission band appears after heterostructure formation with WS_2_, converting into CeO_2_@WS_2_, attributable to the promoted charge separation, which is absent in CeO_2_ alone. These good charge separation and adagio recombination rates between the photo-induced electron-hole pairs are the main origins of the boosted photocatalytic efficiency. Moreover, according to our previous study, the CeO_2_@WS_2_′s bandgap obtained from Tauc’s plots (2.9 eV) [15], [48] is narrower than CeO_2_ (∼3.02 eV) and WS_2_ (3.05 eV) [49]. Lastly, we ought to address the likely lack of contribution from the unintentional CeS phase toward the observed optical properties. CeS has a band gap of ∼ 3.6 eV [50] which is wider than both WS_2_ and CeO_2_′s band gaps [49], falling outside the visible range and, thus, is not likely to contribute to photocatalysis.

**Fig. 2:**
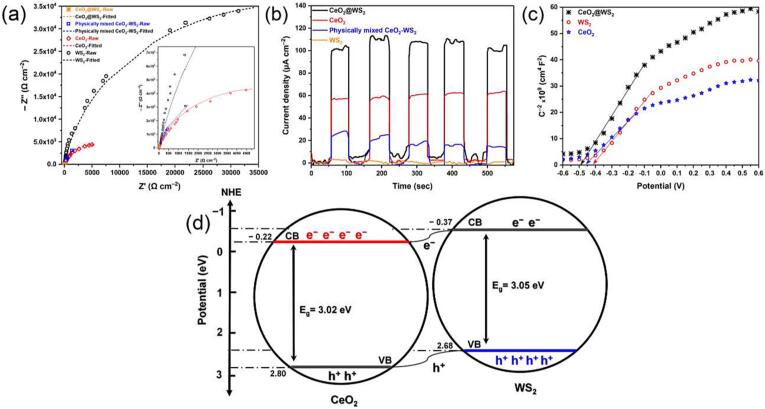
(a) The EIS plots (inset: the magnified form of EIS plots to see details), (b) Photocurrent response, (c) Mott-Schottky plots, and (d) the proposed type (II) heterojunction diagram.

The charge transfer efficiencies of samples were measured by the EIS since it directly influences charge transfer resistance (*R*_ct_). As shown in Fig. 2a, the Nyquist plot of WS_2_ includes a wide semi-circle diameter, demonstrating its higher resistance to charge transfer than other samples. CeO_2_ also has a greater diameter, showing a high charge transfer resistance. However, the CeO_2_@WS_2_ heterostructure revealed a significant reduction in the charge transfer resistance by looking at the diameter of semi-circles which decreases. The *R*_ct_ of WS_2_ was found to be ∼ 35000 Ω cm^−2^, and for CeO_2_, the *R*_ct_ value was ∼ 4500 Ω cm^−2^. Eventually, the CeO_2_@WS_2_ heterostructure’s *R*_ct_ was significantly lower (45 Ω cm^−2^) than both CeO_2_ and WS_2_ (inset of Fig. 2a). Interestingly, the physical mixture of WS_2_ and CeO_2_ showed an *R*_ct_ of 1500 Ω cm^−2^, confirming that the chemically bonded interface of WS_2_ and CeO_2_ plays a substantial role in the electronic structure of the heterostructure. In other words, the charge transport conductivity of the chemically bonded heterostructure (22.23 mS cm^−2^) is by far higher than WS_2_, CeO_2_, and their physical mixture of CeO_2_-WS_2_ (0.67 mS cm^−2^), which confirms the importance of chemical bonding of these two structures at the interface.

The transient photocurrent response *vs* time was measured for each photocatalyst sample as an index of their charge separation and transfer efficiency, as shown in Fig. 2b. In this investigation, the lowest response was observed for WS_2_ inefficient e^−^/h^+^ pairs separation. Albeit, a similar behaviour was obtained for CeO_2_. As predicted, a higher response for CeO_2_@WS_2_ heterostructure was observed, which signifies the heterojunction efficiency in the photoelectrocatalytic performance. However, when WS_2_ was physically mixed with CeO_2_, the photocurrent response had a significantly lower response than even the bare CeO_2_. This observation was further evidence highlighting the role of a stable interface between CeO_2_ and WS_2_ through chemical bonding between Ce and S species.

The Mott-Schottky method helps identify the type of semiconductors (*p* or *n*) and their flat-band energies (*E*_fb_). The Mott-Schottky plots of WS_2_, CeO_2_, and CeO_2_@WS_2_, depicted in Fig. 2c, show all semiconductors are *n*-type due to the potential-C^−2^ plots’ positive slope. Furthermore, the *E*_fb_ can be determined by interrupting the liner part of potential-C^−2^ plots with the potential *x*-axis. The values of *E*_fb_s and our previously reported bandgaps (*E_g_*) [15] for these materials are listed in Table S1. In *n*-type semiconductors, *E*_fb_ can convert to the conduction band (CB) energy using the *E*_CB_ = *E*_fb_ – 0.2 V [51]. Thus, the *E*_CB_s of −0.61, −0.46, and −0.69 V were obtained for WS_2_, CeO_2_, and CeO_2_@WS_2_, respectively. For calculating the valence band (VB) energy, at first, the *E*_CB_ values convert to the normal hydrogen electrode (NHE) values using *E*_NHE_ = *E*_SCE_ + 0.241 V [52]. Then, the *E*_VB_s can be obtainable through the *E*_VB_ = *E*_CB_ + *E*_g_. All described values are listed in Table S1. Considering the CB and VB levels as depicted in Fig. 2d, a plausible type (II) heterojunction can be proposed for the CeO_2_@WS_2_ heterostructure. In such a heterojunction, the photogenerated electrons in WS_2_′s CB tend to migrate to the CeO_2_′s CB and make it the main reactive CB site. In contrast, the photogenerated holes (h^+^) in CeO_2_′s VB migrate to WS_2_′s VB and make it a more reactive VB site [53], [54]. Furthermore, the bandgap narrowing in the CeO_2_@WS_2_ sample in type (II) heterojunction is also justifiable. It is worth noting that the experimentally-obtained bandgap for CeO_2_@WS_2_ through the Tauc equation (2.9 eV) equals a value from CeO_2_′s *E*_CB_ to WS_2_′s *E*_VB_, which further confirms the type II heterojunction [15].

### 3.3 Theoretical insights

Here, we study the interface between CeO_2_ and WS_2_. In constructing our model, we considered the HRTEM image of the CeO_2_@WS_2_ heterostructure in Fig. 1h. We also restricted our models to high-symmetry and low lattice mismatch configurations. To construct the interface at the heterostructure, we first cleaved the WS_2_ hexagonal structure (downloaded from Materials Project [55], compound id: mp-224) along the [0 0 1] direction. We then constructed a supercell with (2*u* ×√2*v*)*R*30° dimensions out of the cleaved surface. The resulting surface had an orthogonal cross-section suitable to be interfaced with cubic CeO_2_ with *u*′ = 6.381 Å and *v*′= 5.527 Å. We then cleave the conventional CeO_2_ structure (*a* = 5.415 Å) along the [0 0 1] direction. We then interfaced these two surfaces together by expanding WS_2_ along the [0 0 1] direction to be three sheets deep and expanding CeO_2_ along the [1 0 0] direction to be 12 atomic layers deep. Since we intended to have only one interface in the supercell, we added an ample vacuum slab of 20 Å to avoid artificial interactions at the non-interfacing facets in the supercell. To reduce the lattice mismatch, we allowed the lateral cell parameters to relax during the geometry optimisation. The lattice mismatch in the relaxed structures had an acceptable average value of ∼ 5.4 %.

**Fig. 3:**
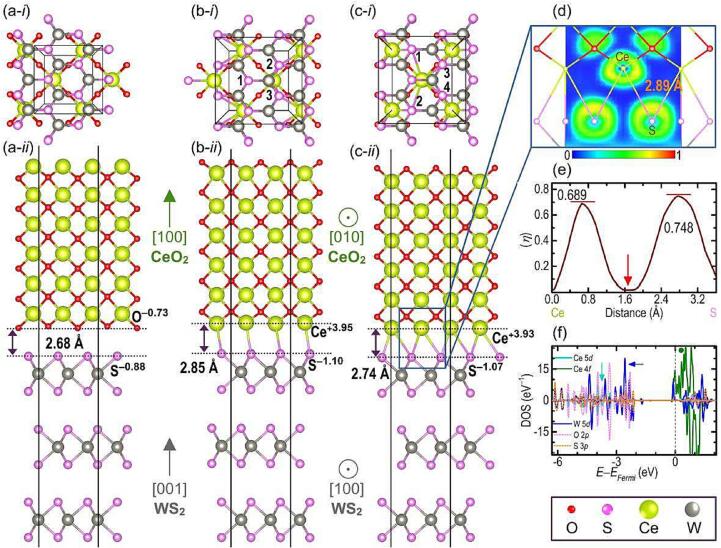
The top (upper rows) and side (lower rows) views of the CeO_2_@WS_2_ interface configurations are shown (a), (b), and (c). The interface in (c) was found to be the most stable. (d) The electronic localisation function (*η*) at the interface region in (c). (e) The *η* line profile of the Ce–S bond at the CeO_2_@WS_2_ interfaces. (f) The partial density of states of the interface shown at (c).

Chemically there are two types of interfaces; either an oxygen-cleaved CeO_2_ facet interfaces with WS_2_, or a Ce-cleaved facet forms the interface with WS_2_. The first case has only one high-symmetry configuration, shown in Fig. 3a. The second scenario has two configurations, shown in Fig. 3b and c, respectively. In Fig. 3b, each Ce ion at the interface is coordinated by 3 S ions, while in Fig. 3c, each Ce ion is coordinated by 4 S ions. The Ce-coordinating S ions are marked in the upper row of Fig. 3. Among these three possible interfaces, configuration C was the most stable with total DFT energy of −531.610 eV, while configurations A and B each had higher total energy of −531.209 and −531.178 eV, respectively. In configuration A, the negatively charged facets at the interface create a Coulombic repulsion that reduces stability—The partial charges borne on S and O ions at the interface are given in Fig. 3a-*ii*. For configuration B, Ce’s undercoordination may be the origin of instability compared to Configuration A, as Ce^4+^ in a compound is more stable when coordinated by eight anions instead of seven [56].

Further examination of the most stable interface of configuration C in Fig. 3c-*ii* shows that the interface separation between CeO_2_ and WS_2_ is 2.74 Å, and the Ce–S bond is 2.93 Å long. This bond length is very similar to the Ce–S bond length in CeS, which is 2.89 Å [57]. Furthermore, the electronic localisation function (*η*) along the Ce–S bond (Fig. 3d, e) indicates two peaks around both Ce and S centres. This *η* profile deviates from a perfect ionic bond where *η* approaches 1 near the anion while approaching 0 near the cation [58]. This profile, however, is not perfectly covalent either. In covalent bonding, *η* is maximum at the bond centre and tapers off at both ends [58]. Consequently, we conclude that the bonding nature at the CeO_2_@WS_2_ interface is only partially ionic (or partially covalent).

The partial density of states of the interfacing layers in Fig. 3f shows that the Ce 4*f* states are all empty and located above the Fermi level (marked with a green circle). However, some of the Ce 5*d* states are still occupied and located below the Fermi level (marked with a cyan arrow), explaining the less than + 4 oxidation state indicated by Bader charge analysis (Fig. 3c-*ii*). Similarly, some of W’s 5*d* states are also below the Fermi level, as marked by a blue arrow, indicating a deviation from the pure ionic + 4 oxidation state. This deviation is probably caused by the covalency between S and W. Partial oxidation of W and S, in turn, raises the Fermi level to the bottom of the conduction band, intercepting W’s 5d states and creating *n*-type carriers, facilitating the observed photocatalytic activity.

### 3.4 Photocatalytic experiments

We studied the impact of the heterostructure in the WS_2_ intercalated CeO_2_ layered materials for the photocatalytic formic acid decomposition. When the heterostructure of two or more different structures is constructive, the photocatalytic activity boosts due to their longer electron/hole lifetime and efficient charge separation. Consequently, the photocatalytic activity improves. The oxidation of formic acid into CO_2_ under photocatalytic conditions is a benchmark reaction that can be useful to judge the photocatalytic capability of the semiconductors that are supposed to be photocatalysts. This photocatalytic test is also suitable for comparing the photocatalysts under identical conditions.

**Fig. 4:**
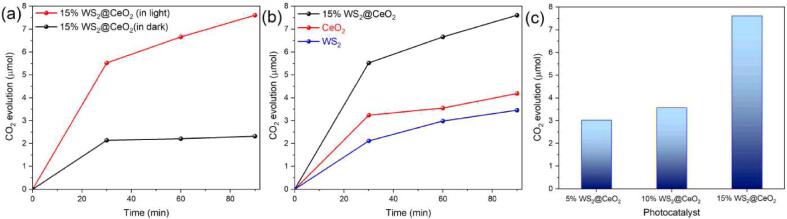
(a) Time-course formic acid decomposition in the presence and absence of light through the catalysis of CeO_2_@WS_2_ (15 wt%). (b) Comparing the photocatalytic activity of the CeO_2_@WS_2_ (15 wt%) with CeO_2_ and WS_2_ nanosheets. (c) Photocatalytic activity of the CeO_2_@WS_2_ with three different amounts of WS_2_ loading, including 5, 10, and 15 wt%. All photocatalytic tests have been performed under the UV–vis range in the aqueous solution of formic acid (5 vol%) in the presence of a catalyst (15 mg) at room temperature.

The photocatalytic activity of CeO_2_@WS_2_ was investigated under UV–vis range irradiation. The formic acid oxidation power of the CeO_2_@WS_2_ was first investigated in the absence and then the presence of light (Fig. 4a). We observed that, within 90 min,  the dispersion of CeO_2_@WS_2_ results in 6.7 µmol (or 446.7 µmol g^-1^) CO_2_ under UV–vis irradiation and 2.2 µmol (146.7 µmol g^-1^) CO_2_ in the dark, three fold higher CO_2_ higher evolution rate originating from light irradiation. After 30 min, when there was no light irradiation, the reaction has minor progress and almost stops while in the presence of light; CO_2_ continued to evolve, further proving that CeO_2_@WS_2_ is photo-catalytically active under UV–vis range irradiation. In the next step, we tried to understand the photoactivity of CeO_2_@WS_2_ under visible light irradiation. Formic acid photooxidation under the visible range is almost similar to dark conditions (Fig. 4a). This observation indicates that the photocatalytic activity is minimal under the visible range. Therefore, we can claim that the photocatalytic activity of CeO_2_@WS_2_ majorly originates from the UV range irradiation.

Lastly, we compared the photocatalytic activity of CeO_2_@WS_2_ with CeO_2_ and WS_2_ on their own (Fig. 4b). We also studied the effect of different amounts of CeO_2_ loading on WS_2_ (Fig. 4c). We realised that CeO_2_ loaded on WS_2_ (15 wt%) causes more photocatalytic activity than the 5 wt% and 10 wt% loading amounts. Accordingly, the CO_2_ production rates for CeO_2_ and WS_2_ were 2.9 µmol and 3.5 µmol in 90 min, respectively, which are significantly lower than the ceria-loaded WS_2_. At last, the produced CO_2_ in this work is comparable to the previous reports [59], [60], [61], *e.g.*, anatase TiO_2_, layered titanate, protonated layered titanate, and 2D layered titanate-based photocatalysts [59], [61]. In another example [60], a newly developed titania-based catalyst (namely, Ti@PMO-Bipy) reports the production of 5.5 µmol CO_2_ in 90 min under optimal conditions while our developed catalyst produces a greater value (6.7 µmol) of CO_2_ within 90 min.

## 4 Conclusions

WS_2_, once exfoliated through ultrasonic waves, could efficiently form chemical bonds with CeO_2_ during the hydrothermal synthesis, creating a promising heterostructure. The chemical bonding between Ce and S at the heterojunction was determined by Raman spectroscopy and XRD. Our density functional calculations could identify the most stable CeO_2_/WS_2_ interface configuration, further confirming the covalence bonding at the interface. Our simulation predicted a 2.74 Å interface separation between phases with a 2.93 Å Ce–S bond length. The density of states at the interface showed the formation of *n*-type carriers, matching with that of Mott-Schottky plot. This ultrasonically-synthesised CeO_2_@WS_2_ heterostructure emerged as a superior photocatalyst compared to WS_2_, CeO_2_, and their physical mixture. Using the Mott-Schottky plots and comparing the *E_g_*s of the heterostructure with the pure phases through the Tauc plots, we showed that the CeO_2_@WS_2_ heterojunction was type (II). We speculate that several other transition metal oxides can be synthesised in the interlayers of WS_2_, further building on the work presented here.

## Declaration of Competing Interest

The authors declare that they have no known competing financial interests or personal relationships that could have appeared to influence the work reported in this paper.

## Data Availability

Data will be made available on request.
