# Supplementary material for: Ultrasonically-assisted synthesis of CeO2 within WS2 interlayers forming type II heterojunction for a VOC photocatalytic oxidation
Source: Ultrason Sonochem. 2022 Nov 28;92:106245. doi: 10.1016/j.ultsonch.2022.106245 (PMC9719093; doi:10.1016/j.ultsonch.2022.106245)
Supplement: Supplementary data 1 [file mmc1.pdf]

Supplementary Data

Ultrasonically-assisted synthesis of CeO_2 within WS_2 interlayers forming type II heterojunction for a VOC photocatalytic oxidation

Esmail Doustkhah,[a,*] Ramin Hassandoost,[b] Negar Yousef Tizhoosh,[b] Mohamed Esmat,[c] Olga Guselnikova,[d] M. Hussein N. Assadi,[e] Alireza Khataee[b,f,*]

[a] *Koç University Tüpraş Energy Center (KUTEM), Department of Chemistry, Koç University, 34450, Istanbul, Turkey*

[b] *Research Laboratory of Advanced Water and Wastewater Treatment Processes, Department of Applied Chemistry, Faculty of Chemistry, University of Tabriz, 51666–16471 Tabriz, Iran*

[c] *International Center for Materials Nanoarchitechtonics (MANA), National Institute for Materials Science (NIMS), 1-1 Namiki, Tsukuba, Ibaraki, 305–0044, Japan*

[d] *Research School of Chemistry & Applied Biomedical Sciences, National Research Tomsk Polytechnic University, Lenin Avenue 30, Tomsk 634050, Russia*

[e] *RIKEN Center for Emergent Matter Science, 2-1 Hirosawa, Wako, Saitama, 351–0198, Japan*

[f] *Department of Environmental Engineering, Gebze Technical University, 41400 Gebze, Turkey*

[*] edoustkhahheragh@ku.edu.tr

[*] a_khataee@tabrizu.ac.ir

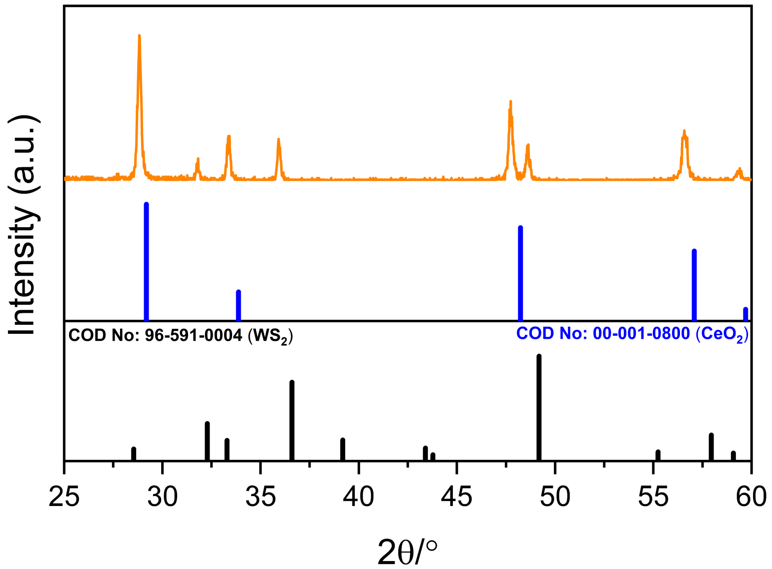

Fig. S1. XRD of physically mixed CeO_2 and WS_2.

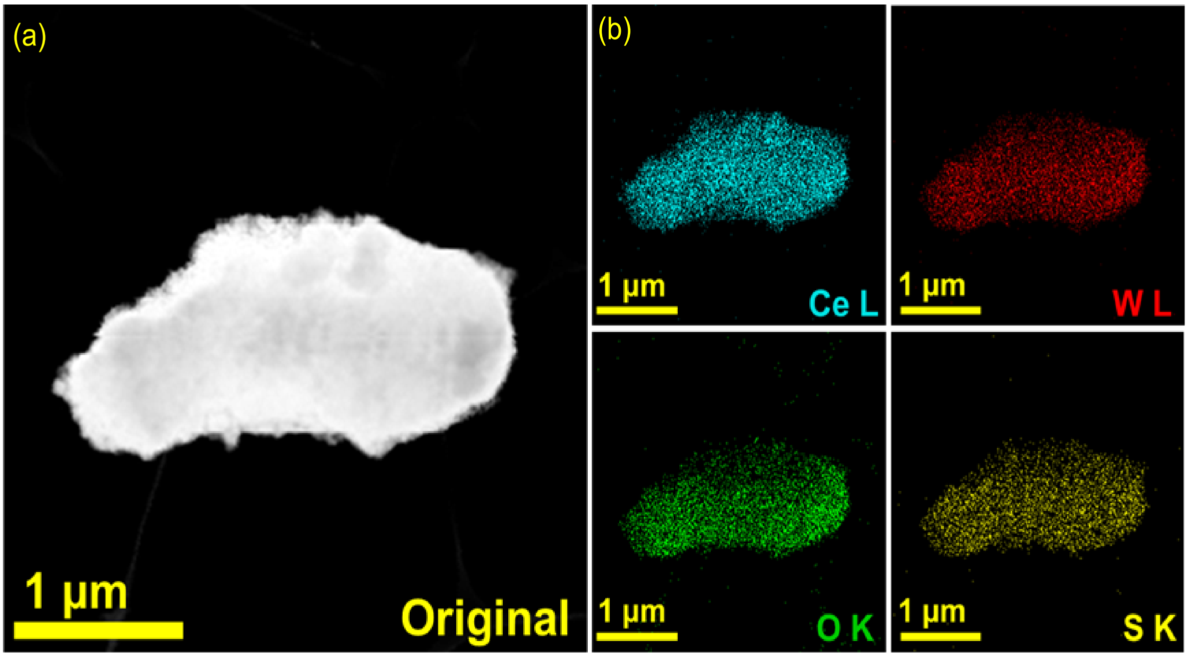

Fig. S2. (a) HAADF-STEM image of a CeO_2@WS_2 particle's. (b) The elemental distribution scanning images.

Table S1. The CB and VB levels obtained from the E_g and E_{fb} values.

Sample	E_{fb} (V)	E_g (eV)	CB (eV)	VB (eV)
**WS_2**	–0.41	3.05	–0.37	2.68
**CeO_2**	–0.46	3.02	–0.22	2.80
**CeO_2@WS_2**	–0.49	2.90	–0.45	2.45
